# Supplementary material for: Discovery of New Triterpenoids Extracted from Camellia oleifera Seed Cake and the Molecular Mechanism Underlying Their Antitumor Activity
Source: Antioxidants (Basel). 2022 Dec 21;12(1):7. doi: 10.3390/antiox12010007 (PMC9854776; doi:10.3390/antiox12010007)
Supplement: Supplementary file 1 [file antioxidants-12-00007-s001.zip › antioxidants-2041883-supplementary.pdf]

Supplementary data

Table S1. Cytotoxic activities of total saponin and total aglycone fractions.

Cell lines	sample	survival %							
		3.125	6.25	12.5	25	50	100	200	ED$_{50}$ (M)
A549	total saponin	82.71%	75.16%	64.60%	49.62%	1.67%	1.67%	2.19%	25.77
		87.93%	62.52%	59.52%	31.38%	1.15%	1.02%	1.41%	
		78.02%	78.81%	63.04%	39.85%	0.11%	0.11%	0.37%	
		71.77%	71.64%	60.43%	39.59%	3.24%	3.63%	3.11%	
		67.47%	60.04%	54.70%	35.29%	1.02%	1.28%	1.67%	
		67.86%	66.04%	48.84%	50.01%	0.89%	0.89%	1.80%	
	total aglycone	93.19%	67.43%	42.99%	1.27%	8.15%	2.15%	1.27%	15.04
		71.09%	36.98%	45.18%	12.69%	10.05%	1.27%	0.83%	
		76.95%	48.55%	41.08%	7.42%	9.90%	0.98%	2.00%	
		73.58%	52.94%	39.03%	19.71%	12.10%	4.78%	3.61%	
		7.86%	57.18%	42.99%	11.08%	10.34%	1.12%	1.56%	
		57.33%	62.45%	44.01%	15.61%	12.10%	2.88%	2.59%	
hela	total saponin	9.24%	67.24%	82.72%	94.26%	61.86%	1.98%	2.61%	56.84
		73.08%	118.44%	52.70%	63.92%	85.41%	1.19%	1.82%	
		58.55%	91.89%	102.95%	77.51%	56.97%	1.03%	0.40%	
		56.33%	67.24%	20.94%	93.47%	74.03%	4.03%	3.71%	
		103.27%	90.62%	95.05%	87.15%	88.73%	1.98%	3.08%	
		134.87%	99.16%	96.63%	104.06%	81.14%	1.19%	1.34%	
	total aglycone	156.20%	79.00%	51.59%	11.61%	7.59%	3.81%	6.83%	4.211
		175.82%	76.99%	33.49%	7.08%	2.31%	2.05%	2.81%	
		170.03%	59.64%	27.70%	6.83%	1.55%	0.54%	2.05%	
		98.62%	69.95%	26.45%	11.36%	12.62%	6.08%	5.83%	
		121.00%	89.06%	23.68%	10.85%	2.81%	2.81%	1.80%	
		130.55%	80.01%	29.21%	11.61%	9.60%	2.56%	2.05%	
HepG2	total saponin	96.84%	84.14%	55.83%	49.55%	22.26%	1.97%	12.04%	37.69
		76.70%	110.27%	99.90%	107.05%	21.53%	2.26%	14.67%	
		92.02%	78.89%	75.68%	93.04%	24.01%	1.82%	8.25%	
		74.22%	77.86%	74.36%	74.36%	15.25%	4.16%	21.82%	
		82.10%	78.45%	73.34%	67.06%	12.48%	1.39%	7.66%	
		83.85%	105.16%	104.28%	81.37%	17.59%	0.07%	7.08%	
	total aglycone	111.21%	88.52%	112.49%	46.30%	27.69%	22.22%	21.17%	18.28
		104.69%	119.35%	98.53%	73.52%	24.08%	20.01%	20.01%	
		94.22%	93.29%	63.51%	30.59%	20.12%	20.01%	19.54%	
		104.81%	98.41%	68.40%	42.11%	20.59%	21.40%	21.64%	
		100.74%	88.64%	68.40%	20.24%	18.96%	19.19%	19.54%	
		103.53%	95.27%	78.87%	51.53%	18.96%	19.19%	23.26%	
